# Impact of exacerbation history on future risk and treatment outcomes in chronic obstructive pulmonary disease patients: A prospective cohort study based on Global Initiative for Chronic Obstructive Lung Disease (GOLD) A and B classifications

**DOI:** 10.7189/jogh.14.04202

**Published:** 2024-10-11

**Authors:** Ling Lin, Qing Song, Wei Cheng, Tao Li, Ping Zhang, Cong Liu, Xueshan Li, Yuqin Zeng, Xin Li, Dan Liu, Yan Chen, Shan Cai, Ping Chen

**Affiliations:** 1 Department of Respiratory and Critical Care Medicine, Second Xiangya Hospital, Central South University, Changsha, China; 2 Research Unit of Respiratory Disease, Central South University, Changsha, China; 3 Clinical Medical Research Centre for Respiratory and Critical Care Medicine, Changsha, China; 4 Diagnosis and Treatment Centre of Respiratory Disease, Central South University, Changsha, China; 5 Division Four of Occupational Diseases, Hunan Prevention and Treatment Institute for Occupational Diseases, Changsha, China; 6 Department of Respiratory Diseases, The Eighth Hospital in Changsha, Changsha, China

## Abstract

**Background:**

In this study, we aimed to explore the impact of exacerbation history on future exacerbation and mortality with different inhaled drugs in chronic obstructive pulmonary disease (COPD) patients based on a Global Initiative Chronic Obstructive Lung Disease (GOLD) A and B classifications.

**Methods:**

This observational study was based on the cohort study Real World Research of Diagnosis and Treatment of COPD (RealDTC). We collected data from COPD patients in China from 1 July 2017 to 31 December 2022. Patients were followed up until December 2023 or death. Further, we separated GOLD A and B patients into GOLD A0 and B0, who had no exacerbation during the previous year, and GOLD A1 and B1, who had only one exacerbation during the previous year. Study outcomes included moderate-to-severe exacerbation, hospitalisation, frequent exacerbation in the first year and all-cause mortality during total follow-up.

**Results:**

Of the 8318 eligible patients, GOLD E group of patients suffered from a greater risk of exacerbation in the first year and death than patients in the GOLD A and B groups. GOLD A1 group had a higher risk of moderate-to-severe exacerbation (hazard ratio (HR) = 2.087; 95% confidence interval (CI) = 1.419–3.068), hospitalisation (HR = 1.704; 95% CI = 1.010–2.705) and frequent exacerbation (HR = 1.983; 95% CI = 1.046–3.709) compared to GOLD A0. GOLD B1 group had a risk of moderate-to-severe exacerbation (HR = 1.321; 95% CI = 1.105–1.679) and mortality (HR = 1.362; 95% CI = 1.026–1.963) that exceeded the risk in GOLD B0 group. The treatment outcome of different inhaled drugs had no statistical differences in GOLD A0 group. In GOLD A1 group, only inhaled corticosteroids (ICS), in addition to long-acting β-2 agonist (LABA) and long-acting muscarinic antagonist (LAMA), reduced the risk of moderate-to-severe exacerbation in the first year compared to only LAMA. As for the GOLD B0 group, LABA and LAMA decreased the odds of moderate-to-severe exacerbation, hospitalisation, frequent exacerbation and mortality compared to only LAMA. ICS, LABA, and LAMA in GOLD B0 also down-regulated the risk of frequent exacerbation, compared to only LAMA. In addition, GOLD B1 patients treated with LABA and LAMA or ICS, LABA, and LAMA had a lower risk of moderate-to-severe exacerbation and hospitalisation. Meanwhile, ICS, LABA, and LAMA also reduced the risk of frequent exacerbation and mortality, compared to only LAMA in the multivariate Cox analysis.

**Conclusions:**

Compared to the GOLD A or B group without exacerbation history, GOLD A patients with exacerbation history had a higher risk of future exacerbation, and GOLD B patients with exacerbation history had a higher risk of future exacerbation and mortality and benefited more from triple inhaler therapy.

Chronic obstructive pulmonary disease (COPD) is a chronic respiratory disease caused by toxic particles or gases [1]. It presents as persistent or repeated respiratory symptoms, including dyspnoea, chest tightness, coughing, and expectoration. Globally, 174.5 million people suffer from COPD, which is the third leading cause of mortality. It was reported that 46% of COPD patients suffered from at least one exacerbation in the preceding year, and 19% required hospitalisation. Exacerbation of COPD can lead to an accelerated decline in lung function, increased mortality or worse quality of life [2–4]. Therefore, it is crucial to choose an effective and appropriate treatment method based on the risk stratification of patients, as well as to effectively evaluate the prognosis.

In the Global Initiative for Chronic Obstructive Lung Disease (GOLD) 2017 report, the GOLD ABCD classification is based on the severity of respiratory symptoms and history of exacerbation but does not include spirometry grades from one to four of the forced expiratory volume in one second percentage predicted (FEV1% predicted) [5]. Although multiple studies have shown that the GOLD ABCD grade has a good predictive value for future exacerbation and death in COPD patients, growing evidence suggests that a history of exacerbation is the best predictor of the risk of exacerbation and death [6]. Recently, GOLD 2023 updated the classification, combining GOLD C and D into one GOLD E group for patients with a history of two or more moderate exacerbations or at least one hospitalisation [1]. Nevertheless, GOLD A and B groups consist of low symptomatic patients and high symptomatic patients with a low risk of future exacerbation, which had less than two exacerbations in the previous year but without hospitalisation.

Patients diagnosed with COPD based on primary and secondary medical insurance in the Norwegian COPD cohort study and from Swedish National Airway Register [7,8], were found to be mostly in the GOLD A and B groups, with GOLD A group accounting for 20–30% and GOLD B group accounting for 40–60% of all patients. Both GOLD A and GOLD B include patients who experienced only one moderate exacerbation in the past year (A1 and B1, respectively) and patients without exacerbation (A0 and B0, respectively). Nevertheless, it has been reflected that compared to COPD patients without history of exacerbation, patients experiencing only one moderate exacerbation had an increased risk of future exacerbation [9]. Further, a prospective study conducted in Denmark including only COPD B patients, indicated that one moderate exacerbation in the preceding year increased the occurrence of subsequent exacerbations and death during the three following years [10].

Existing research and the GOLD E group emphasise the significance of exacerbation history in evaluating prognosis. However, the existing GOLD ABE grade not only provides a basis to evaluate the prognosis but also provides a basis for drug treatment. Moreover, the E group also highlights the importance of the exacerbation history as a reference for inhalation therapy selection. Existing research mainly indicates that long-acting β-2 agonist (LABA) plus long-acting muscarinic antagonist (LAMA) is more beneficial for patients with multiple symptoms than only LAMA or inhaled corticosteroids (ICS) plus LABA [11,12]. Moreover, ICS plus LABA and LAMA is beneficial for patients with frequent exacerbation, or increased eosinophils or asthma [13–15]. However, there is a lack of evidence of inhalation treatment for patients with only one moderate exacerbation. Simultaneously, it is not yet clear whether there is a difference in the treatment effect of different inhalation drugs among patients with and without exacerbation history in perspective with GOLD A and B groups.

In this study, we aimed to compare the risk of future exacerbations and mortality in GOLD A and B patients with and without exacerbation history (GOLD A0 vs A1 and GOLD B0 vs B1). Further, we aimed to additionally explore the clinical outcome (exacerbation and mortality) with different inhaled drugs in GOLD A0, A1, B0, and B1 patients. This will provide realistic evidence for developing more suitable risk stratification to evaluate prognosis and selecting inhalation drugs.

## METHODS

### Study design and subjects

This study was based on the Real World Research of Diagnosis and Treatment of COPD (RealDTC) cohort [16]. RealDTC was a multicentre, prospective cohort study in China conducted from 1 July 2017 to 31 December 2022, and it was assessed retrospectively. Patients were included if they met the diagnosis criteria for COPD defined by the GOLD 2017 recommendations (spirometry with a ratio of the forced expiratory volume in one second (FEV1) to the forced vital capacity (FVC)<0.70, after bronchodilator administration) [5]. Patients were excluded if they had bronchiectasis, pneumonia, or lung cancer and if they experienced exacerbation at the time of screening.

We stratified patients in groups A, B, and E on the basis of symptom burden and history of exacerbation according to the GOLD 2023 guidelines. To distinguish high symptom burden, patients were primarily classified based on COPD assessment test (CAT)≥10 or modiﬁed Medical Research Council (mMRC) score ≥2. In addition, we divided patients with no history of hospitalisation exacerbation and those with one or less moderate exacerbation in the previous year into groups A or B. Those with at least one hospitalisation or at least two moderate exacerbations in the preceding year were divided into group E. We further separated GOLD A and B patients into GOLD A0 and B0, who were without any exacerbation during the previous year, and GOLD A1 and B1, who were with only one moderate exacerbation during the previous year (Table S1 in the **Online Supplementary Document**).

This study was conducted following the Declaration of Helsinki and was approved by the ethics committee of the Second Xiangya Hospital of Central South University. All patients provided written informed consent.

### Data collection and definition

We collected demographic and clinical characteristics at the baseline visit, including age, sex, body mass index (BMI), educational level, smoking history, biofuel and occupational exposure history, pulmonary function (FEV1, FEV1% predicted, and FEV1/FVC), CAT, Clinical COPD Questionnaire (CCQ), mMRC, history of exacerbation (moderate-to-severe) in the previous year and different types of inhaled drugs, including LAMA (tiotropium), ICS and LABA (budesonide/formoterol or salmeterol/fluticasone), LABA and LAMA (indacaterol/glycopyrronium, umeclidinium/vilanterol, or glycopyrronium and formoterol) and ICS, LABA, and LAMA (budesonide/glycopyrronium/formoterol or fluticasone furoate/umeclidinium/vilanterol). All patients received training on the use of inhalation devices on their first visit.

Further, we collected data on moderate-to-severe exacerbation, hospitalisation, all-cause death, and treatment adherence during the visit. We defined moderate exacerbation as exacerbation of respiratory symptoms requiring antibiotics and/or oral corticosteroids. The definition of severe exacerbation was exacerbation requiring hospitalisation or emergency department admission for more than two days during the follow-up period. We also separately analysed hospitalisation, whose definition was the same as that of severe exacerbation. We defined frequent exacerbation as at least two exacerbations during the follow-up. Moderate-to-severe exacerbation included both moderate and severe exacerbation. Future exacerbation included moderate-to-severe exacerbation, hospitalisation, and frequent exacerbation.

### Procedure

According to the research principles, each participant had to receive training before the study, standardise the process, and conduct monthly quality control checks for data collection and follow-up. The clinical physician independently determined the patient’s medication prescription and was unaffected by the study.

We followed up with all participants every six months. We collected the number of exacerbations during that time, the severity of the exacerbation (moderate or severe), survival status, and treatment adherence. The endpoint of follow-up was death, loss of contact, or the last routine follow-up before 31 December 2023.

### Study outcome

The outcomes of this study were moderate-to-severe exacerbation, hospitalisation, frequent exacerbation in the first year, adherence (a proportion of days covered (PDC)≥0.8) [17], and all-cause mortality during total follow-up. PDC was calculated as the total number of days of medication provided divided by the total time.

### Statistical analysis

We used SPSS, version 27.0 (IBM, Armonk, New York, USA) to analyse the data. Continuous variables were expressed as mean (x̄) and standard deviation (SD) or median and interquartile range when appropriate. We tested continuous variables using the student’s *t* test. Otherwise, we applied non-parametric tests to non-normal information. We analysed categorical variables using the χ^2^ test. We used the multivariate Cox regression to compare the risk of future exacerbation and death between groups A0 and A1 and B0 and B1. In addition, with the multivariate Cox regression analysis, we analysed the treatment outcome (future exacerbation or mortality during follow-up) of different inhalation therapies in GOLD groups A0, A1, B0, and B1. We applied hazard ratios (HRs) and 95% confidence intervals (CIs) to the above findings. For all data analyses, a *P*-value <0.05 was considered statistically significant.

## RESULTS

### Baseline characteristics of patients with COPD

**Figure 1:**
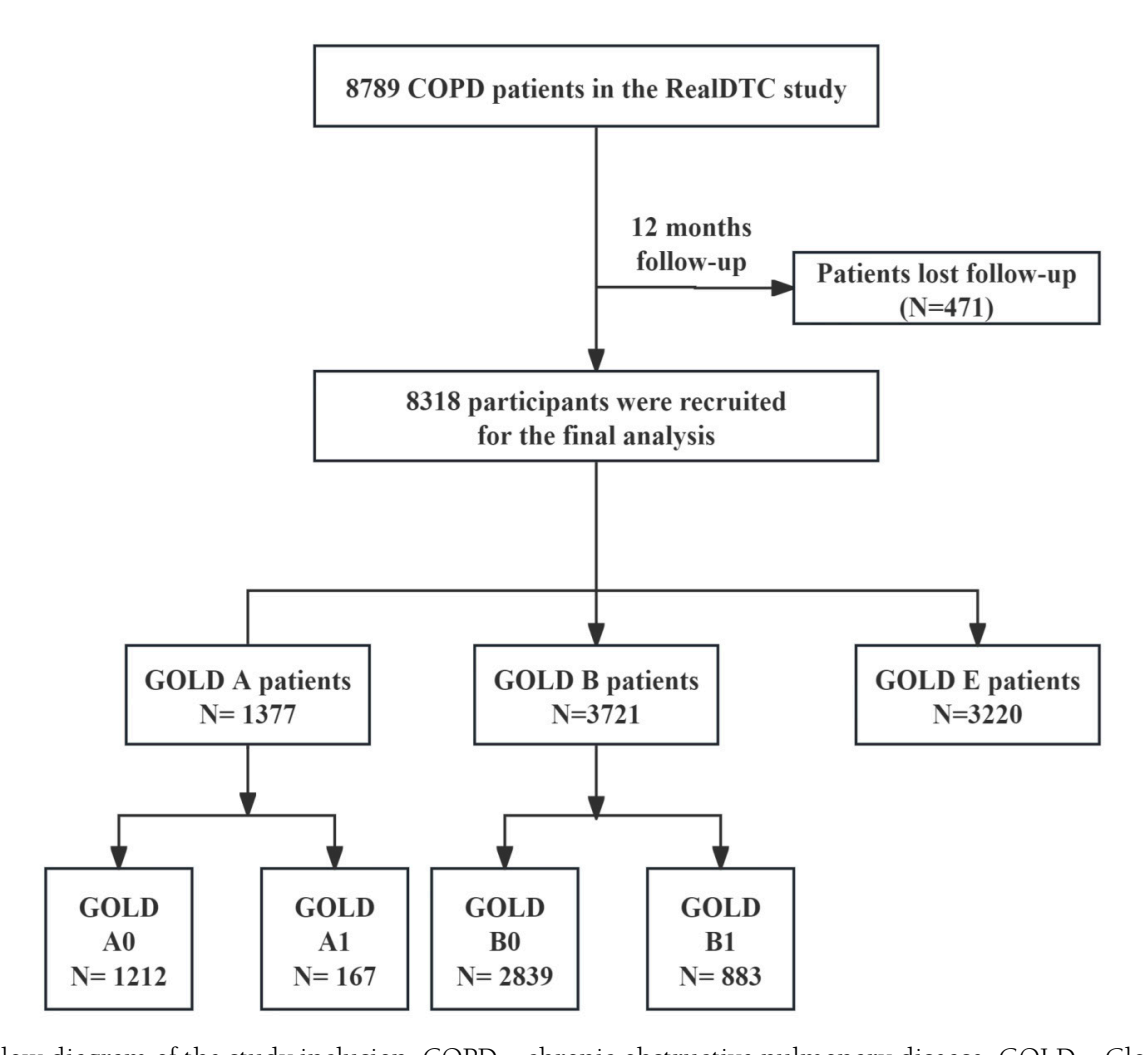
Flow diagram of the study inclusion. COPD – chronic obstructive pulmonary disease, GOLD – Global Initiative for Chronic Obstructive Lung Disease.

A total of 8318 patients with COPD in the RealDTC study were included in this study between 1 July 2017 and 31 December 2022 (**Figure 1**). Of the study population, 14.5% belonged to GOLD group A0, 1.9% to GOLD A1, 34.1% to GOLD B0, 10.7% to GOLD B1, and 38.8% to GOLD E. The average age of all patients was 65.4 years (SD = 9.5), and 85.4% were male. The overall study population mainly consisted of patients with, on average, moderate airflow limitation with FEV1% predicted x̄ = 54.8, SD = 30.1 and FEV1/FVC x̄ = 49.2, SD = 19.8. The x̄ CAT score was 14.3 (SD = 6.8), and the CCQ score was 20.1 (SD = 7.7). Compared with the GOLD A0 group, GOLD A1 had higher CAT and CCQ scores but lower FEV1% predicted and FEV1/FVC. Compared with the GOLD B0 group, GOLD B1 had higher CAT and CCQ scores, but lower FEV1% predicted (**Table 1**).

**Table 1:** Baseline characteristics of the study population*

Variables	Total	GOLD A0	GOLD A1	GOLD B0	GOLD B1	GOLD E	*P*-value
Participants	8318 (100)	1212 (14.5)	165 (2.0)	2839 (34.1)	882 (10.7)	3220 (38.7)	
Age in years, x̄ (SD)	65.4 (9.5)	61.5 (10.8)	62.8 (10.0)	65.7 (9.0)	65.6 (9.8)	66.6 (8.9)	<0.001
Male	7105 (85.4)	1038 (85.6)	142 (86.1)	2447 (86.2)	733 (83.1)	2745 (85.2)	
Education							<0.001
*Under junior high school*	6531 (78.6)	632 (66.6)	118 (71.6)	2224 (78.4)	737 (83.5)	2646 (82.2)	
*Over high school*	1787 (21.4)	406 (33.4)	47 (28.4)	615 (21.6)	145 (16.5)	574 (17.8)	
Married	7841 (94.4)	1143 (95.4)	158 (95.7)	2706 (95.4)	841 (95.4)	2996 (93.1)	0.868
BMI in kg/m^2^, x̄ (SD)	22.7 (3.5)	23.2 (3.0)	23.2 (3.0)	22.8 (3.4)	22.5 (3.5)	22.4 (3.5)	<0.001
Smoking status							0.324
*Smoker*	6201 (74.5)	882 (72.8)	124 (75.2)	2119 (74.6)	617 (70.0)	2459 (76.4)	
*Non-smoker*	2117 (25.5)	330 (27.2)	41 (24.8)	720 (25.4)	265 (30.0)	761 (23.6)	
Biofuel exposure	2940 (35.4)	284 (23.4)	38 (23.0)	932 (32.8)	271 (30.7)	1415 (43.9)	0.160
Occupational exposure	2920 (35.1)	430 (35.5)	62 (37.6)	939 (33.1)	295 (33.4)	1194 (37.1)	0.142
CAT, x̄ (SD)	14.3 (6.8)	5.9 (2.4)	6.4 (2.1)	15.1 (5.7)	16.2 (5.5)†	16.7 (6.6)	<0.001
CCQ, x̄ (SD)	20.1 (7.7)	10.9 (4.9)	12.1 (4.5)‡	20.1 (6.3)	22.1 (6.2)†	23.3 (6.9)	<0.001
FEV1% predicted, x̄ (SD)	54.8 (30.1)	74.6 (19.3)	68.0 (20.2)‡	55.9 (20.0)	53.2 (19.6)†	51.8 (27.5)	<0.001
FEV1/FVC, x̄ (SD)	49.2 (19.8)	58.9 (9.4)	55.2 (11.3)‡	47.9 (12.0)	48.9 (12.0)	46.1 (15.8)	<0.001
Hypertension	241 (2.9)	51 (4.2)	8 (4.8)	71 (2.5)	20 (2.2)	88 (2.7)	0.079
Diabetes	92 (1.1)	10 (0.9)	2 (1.2)	29 (1.0)	10 (1.1)	41 (1.3)	0.765
Cardiovascular diseases	158 (1.9)	13 (1.1)	1 (0.6)	37 (1.3)	17 (1.9)	90 (2.8)	0.237

BMI – body mass index, CAT – chronic obstructive pulmonary disease assessment test, CCQ – clinical chronic obstructive pulmonary disease questionnaire, COPD – chronic obstructive pulmonary disease, FEV1 – forced expiratory volume in one second, FVC – forced vital capacity, GOLD – Global Initiative for Chronic Obstructive Lung Disease, mMRC – modified Medical Research Council dyspnoea scale, SD – standard deviation, x̄ – mean

*Presented as n (%) unless specified otherwise.

†*P* < 0.05 when compared with GOLD B0.

‡*P* < 0.05 when compared with GOLD A0.

### Risk of future exacerbation and mortality for the GOLD A1 vs A0 and GOLD B1 vs B0

**Figure 2:**
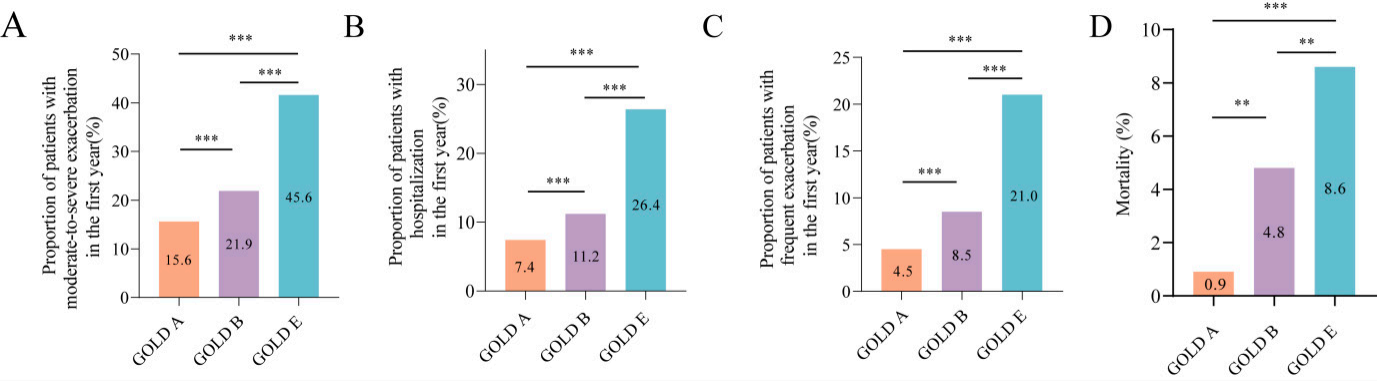
The incidence of future exacerbation and death during follow-up in groups A, B and E. **Panel A.** Comparison for incidence of moderate-to-severe exacerbation between GOLD A, B, and E groups. **Panel B.** Comparison of incidence of hospitalisation between GOLD A, B, and E groups. **Panel C.** Comparison of incidence of frequent exacerbation between GOLD A, B, and E groups. **Panel D.** Comparison of mortality between GOLD A, B, and E groups. GOLD – Global Initiative for Chronic Obstructive Lung Disease.

The proportion of patients with good medication adherence with PDC≥0.8 was 72.3% during follow-up (**Table 2**). Of the eligible patients, 2360 (28.4%) patients had at least one moderate-to-severe exacerbation, 1370 (16.5%) experienced hospitalisation, and 1058 (12.7%) experienced frequent exacerbation during the first-year follow-up. Moreover, the mortality was 5.6%. GOLD E group had a higher incidence of moderate-to-severe exacerbation, hospitalisation, and frequent exacerbation in the first year compared to the GOLD A and B groups (**Figure 2**). The multivariate Cox analysis reflected that the risk of moderate-to-severe exacerbation, hospitalisation, and frequent exacerbation in the GOLD E group exceeded that in the GOLD A and B groups after adjusting for age, gender, BMI, education, smoking status, comorbidities, FEV1% predicted, and CAT (**Table 3**).

**Table 2:** Treatment adherence and the incidence of future exacerbation and death during follow-up in groups A0, A1, B0, B1, and E, presented as n (%)

Variables	Total (n = 8318)	GOLD A0 (n = 1212)	GOLD A1 (n = 165)	GOLD B0 (n = 2839)	GOLD B1 (n = 882)	GOLD E (n = 3220)	*P*-value
Adherence (PDC≥0.8)	6009 (72.3)	846 (69.8)	116 (70.2)	2058 (72.5)	636 (72.2)	2353 (73.1)	0.309
Moderate-to-severe exacerbation	2360 (28.4)	172 (14.2)	41 (24.8)*	576 (20.3)*	225 (25.5)*†	1349 (41.9)*	<0.001
Hospitalisation	1370 (16.5)	83 (6.8)	19 (11.5)*	318 (11.2)*	99 (11.2)*	849 (26.4)*	<0.001
Frequent exacerbation	1058 (12.7)	49 (4.0)	13 (7.9)*	245 (8.6)*	75 (8.5)*	676 (21.0)*	<0.001
Mortality	468 (5.6)	7 (0.6)	4 (2.4)	120 (4.3)*	59 (6.6)*†	276 (8.5)*	<0.001

GOLD – Global Initiative for Chronic Obstructive Lung Disease, PDC – proportion of days covered

*P < 0.05 when compared with GOLD A0.

†*P* < 0.05 when compared with GOLD B0.

**Table 3:** Hazard ratios for future exacerbation and mortality for GOLD A, B, and E groups*

	Moderate-to-severe exacerbation		Hospitalisation		Frequent exacerbation		Mortality	
**Group**	**HR (95% CI)**	***P*-value**	**HR (95% CI)**	***P*-value**	**HR (95% CI)**	***P*-value**	**HR (95% CI)**	***P*-value**
A	Ref.		Ref.		Ref.		Ref.	
B	1.401 (1.142–1.807)	<0.001	1.236 (0.981–1.753)	0.098	1.885 (1.145–2.598)	0.007	2.205 (1.161–3.984)	0.018
E	2.573 (1.967–3.303)	<0.001	2.687 (1.892–3.559)	<0.001	3.387 (2.589–4.531)	<0.001	3.418 (1.856–5.337)	<0.001

CI – confidence interval, GOLD – Global Initiative for Chronic Obstructive Lung Disease, HR – hazard ratio, ref – reference

*Age, gender, education, BMI, smoking status, comorbidities, FEV1% predicted, CAT, and PDC were included as the variables in the multivariate Cox analysis.

Patients in the GOLD A1 group had a higher incidence of moderate-to-severe exacerbation (24.8% vs 14.2%, *P* < 0.001), hospitalisation (11.5% vs 6.8%, *P* = 0.032) and frequent exacerbation (7.9% vs 4.0%, *P* = 0.026) than GOLD A0 group. The mortality between the two groups was similar. Compared with the GOLD B0 group, more patients in the GOLD B1 experienced moderate-to-severe exacerbation and death during visits, but the probability of hospitalisation and frequent exacerbation between these two groups were similar (**Table 2**).

**Figure 3:**
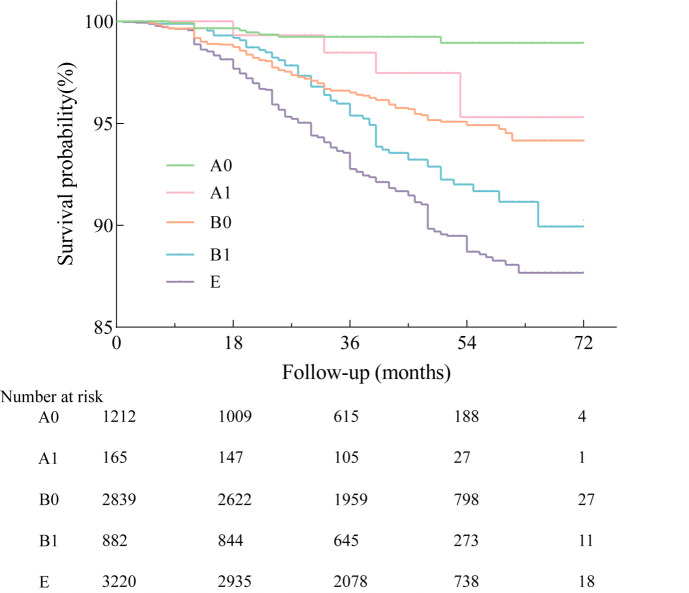
The Kaplan-Meier graphs for mortality in GOLD A1, A1, B0, B1, E.

After adjusting for age, gender, BMI, education, smoking status, comorbidities, FEV1% predicted, and CAT in the multivariate Cox analysis, the GOLD A1 group had an increased risk of moderate-to-severe exacerbation (HR = 2.087; 95% CI = 1.419–3.068, hospitalisation (HR = 1.704; 95% CI = 1.010–2.705), and frequent exacerbation (HR = 1.983; 95% CI = 1.046–3.709) compared to the GOLD A0 (**Table 4**). Further, the GOLD B1 group suffered from a greater risk of moderate-to-severe exacerbation (HR = 1.321; 95% CI = 1.105–1.679) and mortality (HR = 1.362; 95% CI = 1.026–1.963) compared to the GOLD B0, except for hospitalisation and frequent exacerbation (**Table 5**). The multivariate Cox analysis also displayed that the risk of hospitalisation, frequent exacerbation and mortality gradually increased by GOLD groups in HR value, except for moderate-to-severe exacerbations, which were higher in A1 than in B0 (**Figure 3**, Table S2 in the **Online Supplementary Document**). Furthermore, whether during the coronavirus disease 2019 (COVID-19) period or the non-COVID-19 period, the multivariate regression analysis indicated that group A1 had a higher risk of exacerbation than groups A0 and group B1 had a greater risk of exacerbation than group B0. Further, group B1 had a higher risk of death than B0 during the COVID-19 pandemic compared to the non-COVID-19 period (TablesS4–7 in the **Online Supplementary Document**).

**Table 4:** Hazard ratios for exacerbation and mortality for GOLD A1 vs A0*

	Moderate-to-severe exacerbation		Hospitalisation		Frequent exacerbation		Mortality	
**Group**	**HR (95% CI)**	***P*-value**	**HR (95% CI)**	***P*-value**	**HR (95% CI)**	***P*-value**	**HR (95% CI)**	***P*-value**
A0	Ref.		Ref.		Ref.		Ref.	
A1	2.087 (1.419–3.068)	<0.001	1.704 (1.010–2.705)	0.045	1.983 (1.046–3.709)	0.036	2.213 (0.775–9.087)	0.134

CI – confidence interval, GOLD – Global Initiative for Chronic Obstructive Lung Disease, HR – hazard ratio, ref – reference

*Age, gender, education, BMI, smoking status, comorbidities, FEV1% predicted, CAT, and PDC were included as the variables in the multivariate Cox analysis.

**Table 5:** Hazard ratios for exacerbation and mortality for GOLD B1 vs B0*

	Moderate-to-severe exacerbation		Hospitalisation		Frequent exacerbation		Mortality	
**Group**	**HR (95% CI)**	***P*-value**	**HR (95% CI)**	***P*-value**	**HR (95% CI)**	***P*-value**	**HR (95% CI)**	***P*-value**
B0	Ref.		Ref.		Ref.		Ref.	
B1	1.321 (1.105–1.679)	0.003	0.985 (0.773–1.286)	0.652	0.973 (0.799–1.421)	0.473	1.362 (1.026–1.963)	0.041

CI – confidence interval, GOLD – Global Initiative for Chronic Obstructive Lung Disease, HR – hazard ratio, ref – reference

*Age, gender, education, BMI, smoking status, comorbidities, FEV1% predicted, CAT, and PDC were included as the variables in the multivariate Cox analysis.

### The future exacerbation and mortality in groups A0, A1, B0, B1 with different inhaled drugs

**Figure 4:**
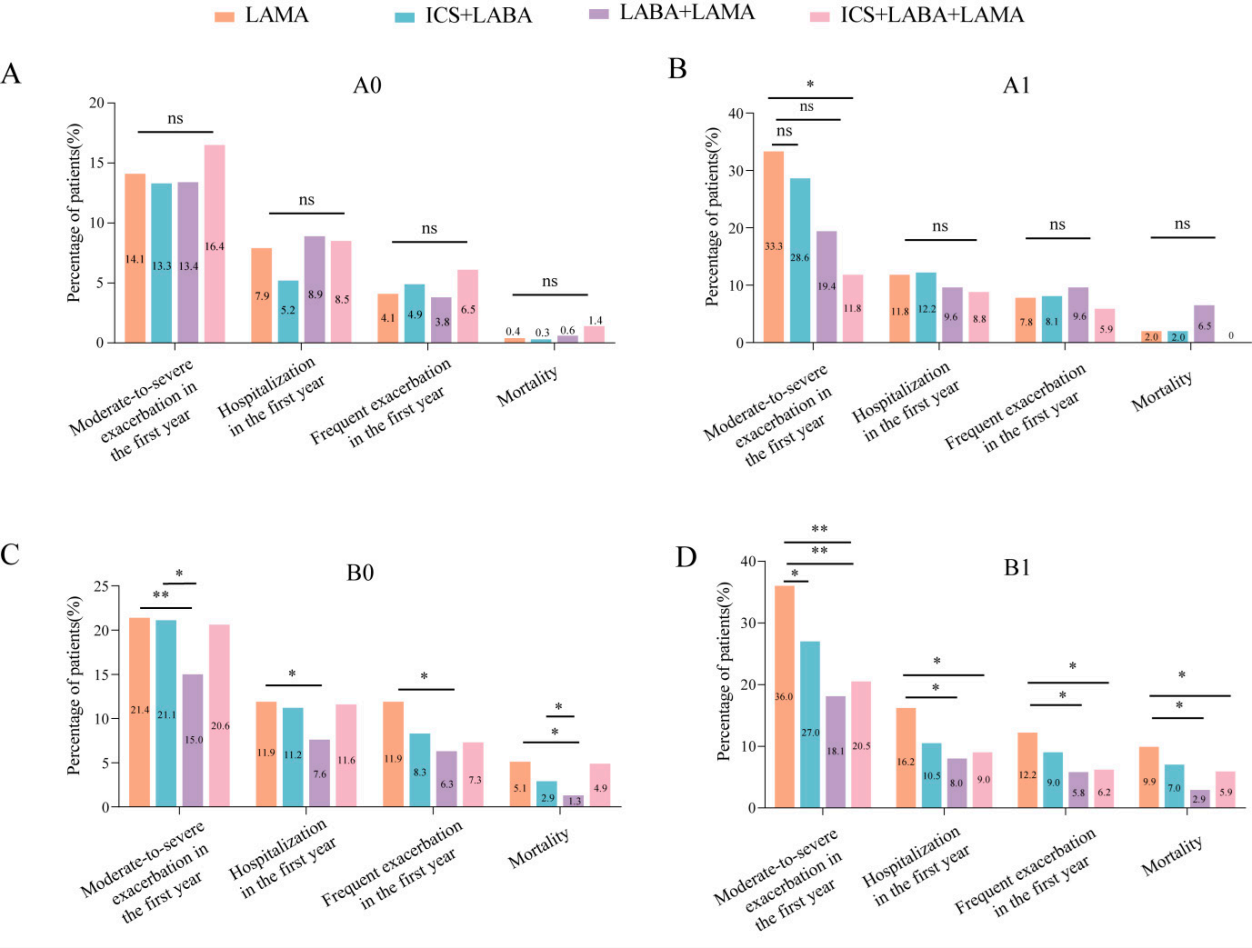
The future exacerbation and mortality in GOLD A0, A1, B0, B1 with different inhalation drugs. **Panel A.** The rate of future exacerbation and mortality in GOLD A0. **Panel B.** The rate of future exacerbation and mortality in GOLD A1. **Panel C.** The rate of future exacerbation and mortality in GOLD B0. **Panel D.** The rate of future exacerbation and mortality in GOLD B1. ICS – inhaled corticosteroids, LABA – long-acting β-2 agonist, LAMA – long-acting muscarinic antagonist.

The incidence of future exacerbation and mortality was similar in patients with different inhalation drugs in group A0 (**Figure 4**). The incidence of moderate-to-severe exacerbation in patients treated with ICS, LABA, and LAMA was lower than in patients treated with LAMA only in the A1 group. In group B0, fewer patients with LABA and LAMA therapy experienced moderate-to-severe exacerbation (15.1% vs 21.4), hospitalisation (7.6% vs 11.9), and frequent exacerbation (6.3% vs 11.9) compared to only LAMA therapy during the visit. Patients with LABA and LAMA therapy had a lower incidence of moderate-to-severe exacerbation than patients with ICS and LAMA therapy (*P* = 0.031). In addition, a lower proportion of patients treated with ICS and LABA or LABA and LAMA died compared to patients treated with LAMA therapy during follow-up. In group B1, patients treated with LABA and LAMA or ICS, LABA, and LAMA had a lower incidence of moderate-to-severe exacerbation, hospitalisation, frequent exacerbation and mortality than patients treated with LAMA only. Otherwise, fewer patients treated with ICS and LAMA experienced moderate-to-severe exacerbation than compared to patients treated with LAMA during follow-up (*P* = 0.046).

Using the multivariate Cox analysis, we exhibited no risk of future exacerbation and mortality in GOLD A0 group with different inhalation drugs (**Table 6**). In GOLD A1 group, patients treated with ICS, LABA, and LAMA had a lower risk of moderate-to-severe exacerbation than patients treated with LAMA when adjusted for age, gender, BMI, education, smoking status, comorbidities, FEV1% predicted, and CAT. The risk of exacerbation and death of patients treated with LABA and LAMA, as well as only LAMA, had no discrepancies. As for patients in the GOLD B0 group, in the multivariate Cox analysis, LABA plus LAMA therapy reduced the risk of moderate-to-severe exacerbation, hospitalisation, frequent exacerbation and mortality compared to treatment with LAMA only. In addition, ICS, LABA, and LAMA down-regulated the risk of frequent exacerbation compared to LAMA. Moreover, compared with ICS and LABA, LABA and LAMA therapy also decreased the risk of moderate-to-severe exacerbation (Table S3 in the **Online Supplementary Document**). In GOLD B1, the multivariate Cox analysis reflected that patients treated with LABA and LAMA therapy had a lower risk of moderate-to-severe exacerbation and hospitalisation than patients treated with LAMA (after adjusting for age, gender, BMI, education, smoking status, comorbidities, FEV1% predicted, and CAT). Moreover, the GOLD B1 group receiving ICS, LABA, and LAMA reduced moderate-to-severe exacerbation, hospitalisation, frequent exacerbation, and mortality compared to those receiving only LAMA. The treatment outcome of different inhaled drugs in the COVID-19 pandemic and non-COVID-19 pandemic was similar in different GOLD groups (Tables S8–9 in the **Online Supplementary Document**).

**Table 6:** Hazard ratios of different inhalation drugs for future exacerbation and mortality in groups A0, A1, B0, and B1

	Moderate-to-severe exacerbation				Hospitalisation			Frequent exacerbation				Mortality			
**Group and inhalation drug**	**HR (95% CI)**		***P*-value**		**HR (95CI%)**		***P*-value**	**HR (95CI%)**			***P*-value**	**HR (95CI%)**			***P*-value**
A0																
*LABA*	Ref.			Ref.					Ref.				Ref.			
*ICS+LABA*	0.924 (0.627–1.386)	0.762		1.114 (0.596–1.642)		0.711			1.053 (0.784–1.423)	0.763			1.098 (0.011–6.486)	0.968		
*LABA+LAMA*	0.933 (0.588–1.592)	0.782		0.976 (0.821–1.469)		0.563			1.122 (0.684–1.742)	0.838			2.621 (0.238–9.362)	0.662		
*ICS+LABA+LAMA*	1.048 (0.689–1.536)	0.578		0.990 (0.678–1.398)		0.875			1.013 (0.667–1.875)	0.646			3.465 (0.757–10.869)	0.187		
A1																
*LABA*	Ref.			Ref.					Ref.				Ref.			
*ICS+LABA*	0.818 (0.335–1.996)	0.659		0.909 (0.477–2.641)		0.361			1.191 (0.150–4.439)	0.869			1.021 (0.110–6.982)	0.970		
*LABA+LAMA*	1.043 (0.371–2.935)	0.936		0.928 (0.238–3.337)		0.806			0.912 (0.420–2.523)	0.230			1.063 (0.203–8.336)	0.672		
*ICS+LABA+LAMA*	0.277 (0.083–0.930)	0.038		0.945 (0.237–3.751)		0.848			1.117 (0.240–3.320)	0.540			0.927 (0.511–2.479)	0.378		
B0																
*LABA*	Ref.			Ref.					Ref.				Ref.			
*ICS+LABA*	1.004 (0.760–1.328)	0.976		0.942 (0.658–1.348)		0.743			0.853 (0.530–1.373)	0.513			0.417 (0.203–0.856)	0.017		
*LABA+LAMA*	0.69 (0.453–0.932)	0.019		0.607 (0.376–0.979)		0.041			0.416 (0.218–0.796)	0.008			0.209 (0.062–0.713)	0.012		
*ICS+LABA+LAMA*	0.935 (0.751–1.165)	0.551		0.950 (0.719–1.254)		0.711			0.659 (0.447–0.970)	0.035			0.787 (0.508–1.218)	0.283		
B1																
*LABA*	Ref.			Ref.					Ref.				Ref.			
*ICS+LABA*	0.770 (0.445–1.334)	0.351		0.459 (0.205–1.028)		0.058			0.713 (0.312-1.629)	0.422			0.415 (0.140–1.233)	0.113		
*LABA+LAMA*	0.400 (0.212–0.753)	0.005		0.532 (0.235–0.987)		0.040			0.395 (0.145–1.072)	0.068			0.339 (0.089–1.279)	0.110		
*ICS+LABA+LAMA*	0.448 (0.266–0.755)	0.003		0.463 (0.230–0.933)		0.031			0.337 (0.146–0.776)	0.011			0.257 (0.083–0.796)	0.019		

GOLD – Global Initiative for Chronic Obstructive Lung Disease, HR – hazard ratio, ICS – inhaled corticosteroids, LABA – long-acting β-2 agonist, LAMA – long-acting muscarinic antagonist, ref – reference

*Age, gender, education, BMI, smoking status, comorbidities, FEV1% predicted, CAT, and PDC were included as the variables in the multivariate Cox analysis.

## DISCUSSION

Growing research and the GOLD ABE classification emphasise the significance of exacerbation history in evaluating prognosis [1,18]. Our study was a real-world observational study aimed to compare the clinical outcomes, including future exacerbation and mortality, in COPD patients who were classified as GOLD A and B group on the basis of the presence and absence of an exacerbation in the past year.

The result indicated that the GOLD E group suffered a greater risk of exacerbation in the first year and death than GOLD A and B, consistent with the current studies and GOLD recommendations [1,19,20]. We discovered that GOLD A and B patients with one moderate exacerbation in the previous year (GOLD A1 and B1) experienced a higher risk of exacerbation during the first-year follow-up or mortality compared to the GOLD A and B patients without exacerbation in the previous year (GOLD A0 and B0). In general, the risk of hospitalisation, frequent exacerbation and mortality was raised by the groups (A0-A1-B0-B1-E), but the odds of moderate-to-severe exacerbation in group A1 exceeded that in group B0. This result was similar to a cohort study based on the Swedish National Airway Register in Sweden, in which GOLD A1 and B1 groups had a higher risk of future exacerbation and respiratory hospitalisations, but not mortality, compared to GOLD A0 and B0, even if the patient comes from a different continent [21]. This may reflect that groups A1 and A0, as well as B1 and B0, have different risk stratification. This will prompt reflection on why we, according to the current treatment recommendation, must wait until the patient has suffered more than one exacerbation or hospitalisation to initiate effective preventive or upgraded treatment.

Most COPD patients are in the GOLD A and B groups, and the present study reflected that the GOLD A1 and B1 groups suffered from a higher risk of future exacerbation. GOLD A0 and B0 patients seem to have a much lower risk for exacerbations. COPD exacerbation can lead to worse respiratory symptoms, accelerated decline in lung function, increased mortality, and more medical costs. Others have reported a reduced risk of exacerbation with triple inhaler therapy compared with dual bronchodilator therapy, even in patients with only one exacerbation in the past year [14,22,23]. Based on the above, our findings argue against the current recommendations that patients have a similar risk profile and should be treated in the same way as the GOLD A and B patients with or without an exacerbation history.

We further explored the clinical outcomes with different inhaled drugs in GOLD A0, A1, B0, and B1 patients. GOLD A0 patients with different inhalation drugs had no difference in risk of future exacerbation and mortality. In GOLD A1, treatment with ICS, LABA, and LAMA had a lower risk of moderate-to-severe exacerbation than treatment with LAMA. However, LABA and LAMA treatment did not decrease the risk of exacerbation and death compared to only LAMA treatment in group A1. The possible reason is that the sample size in group A1, or even the number of cases in group A1 of LABA and LAMA treatment, was small, which may affect the statistical result. As for patients in the GOLD B0 group, LABA and LAMA therapy reduced the risk of future exacerbation and mortality compared to only LAMA. ICS, LABA, and LAMA treatment also down-regulated the risk of frequent exacerbation compared to treatment with LAMA in the GOLD B0 group. In addition, in the multivariate Cox analysis, GOLD B1 patients treated with both LABA and LAMA had a lower risk of moderate-to-severe exacerbation and hospitalisation, while treatment with ICS, LABA, and LAMA reduced the odds of moderate-to-severe exacerbation, hospitalisation, frequent exacerbation, and mortality compared to LAMA treatment. These results reflected that triple inhaler therapy reduced the risk of exacerbation or mortality for patients with one exacerbation but no hospitalisation in the last year (GOLD A1 and B1 groups). However, we took the lead in analysing the association between clinical outcome and perspective with patients with different inhalation drugs in GOLD A and B groups with and without one moderate exacerbation in the previous year. The results were not contradictory to others, in which triple inhaler therapy reduced the risk of exacerbation in patients with only one exacerbation in the past year [23]. Moreover, these findings object to the current recommendations that only patients who suffered more than one exacerbation or at least one hospitalisation in the previous year should escalate therapy to prevent future exacerbation. This will provide realistic evidence for making more suitable risk stratification to evaluate prognosis and select appropriate inhalation drugs. The result of our study also supports that LAMA treatment was sufficient for the GOLD A0 and GOLD B0 patients benefited more from dual bronchodilator therapy.

It’s reported that COVID-19 influenced the exacerbation and mortality of COPD patients. Although COPD has been associated with increased severity and mortality of COVID-19 [24], several studies presented that the risk of hospitalisation is reduced due to the reduced crowd gathering during the COVID-19 period [25]. Therefore, we explored the impact of COVID-19 on the treatment outcome. The PDC and incidence of future exacerbation in the COVID-19 pandemic and non-COVID-19 period had no significant difference. However, there was a lower mortality during the COVID-19 pandemic. Furthermore, COVID-19 did not affect the treatment outcome of different inhaled drugs in different GOLD groups.

Our study has a few potential limitations. First, due to the lower proportion of exacerbation history in GOLD A patients who were with low symptoms, the number of cases in group A1 in our study was relatively small, which may affect the result that compared the clinical outcomes of GOLD A1 patients with different inhalation drugs. Second, although we adjusted for different potential confounders in this real-world study, residual confounding might have affected our results, such as depression, environmental exposures, physical activity, diet, and adherence to other prescribed therapies. Third, the study is based on a Chinese cohort, which may affect the applicability of the results to the broader population because of differences in health care systems, genetic backgrounds, environmental factors, etc. Since we used an ongoing prospective design, further studies can be carried out.

## CONCLUSIONS

Patients in the GOLD A group with exacerbation history had a higher risk of future exacerbation, and patients in the GOLD B group with exacerbation history had a higher risk of future exacerbation and mortality compared to the patients in the GOLD A or B groups without exacerbation history. Further, patients in the GOLD A and B groups with exacerbation history benefited more from triple inhaler therapy. Stratification of GOLD A and B patients with or without a history of exacerbation in the previous year provides valuable information on future risks and the treatment effectiveness of different inhaled drugs, which may affect treatment recommendations for improving prognosis.

## Additional material


Online Supplementary Document
