# Supplementary material for: Impact of exacerbation history on future risk and treatment outcomes in chronic obstructive pulmonary disease patients: A prospective cohort study based on Global Initiative for Chronic Obstructive Lung Disease (GOLD) A and B classifications
Source: J Glob Health. 2024 Oct 11;14:04202. doi: 10.7189/jogh.14.04202 (PMC11466499; doi:10.7189/jogh.14.04202)
Supplement: Online Supplementary Document [file jogh-14-04202-s001.pdf]

Supplemental Table 1. The definition of COPD GOLD group.

Group	Symptom	Exacerbation history
A	mMRC < 2 and CAT < 10	≤1 moderate exacerbation， not leading to hospital admission
A0	mMRC < 2 and CAT < 10	Without exacerbation
A1	mMRC < 2 and CAT < 10	1 moderate exacerbation
B	mMRC≥2 or/and CAT≥10	≤1 moderate exacerbation， not leading to hospital admission
B0	mMRC≥2 or/andCAT≥10	Without exacerbation
B1	mMRC≥2 or/and CAT≥10	1 moderate exacerbation
E	mMRC≥2 or/and CAT≥10	≥2 moderate exacerbation or ≥ 1 leading to hospitalization

Abbreviations: COPD, Chronic Obstructive Pulmonary Diseas; CAT, COPD Assessment Test; GOLD, Global Initiative for Chronic Obstructive Lung Disease; mMRC, modified medical research council dyspnea scale.

Supplemental Figure 1. The incidence of future and mortality during during COVID-19 pandemic and non-during COVID-19 pandemic.(A)Comparison of the incidence of future and mortality during during COVID-19 pandemic and non-during COVID-19 pandemic;(B)PDC of patients during COVID-19 pandemic and non-during COVID-19 pandemic.(C) Comparison of the incidence of future and mortality during during COVID-19 pandemic and non-during COVID-19 pandemic in GOLD A0,A1,B0,B1 groups.

Abbreviations: COVID-19,coronavirus disease 2019; PDC, proportion of days covered.

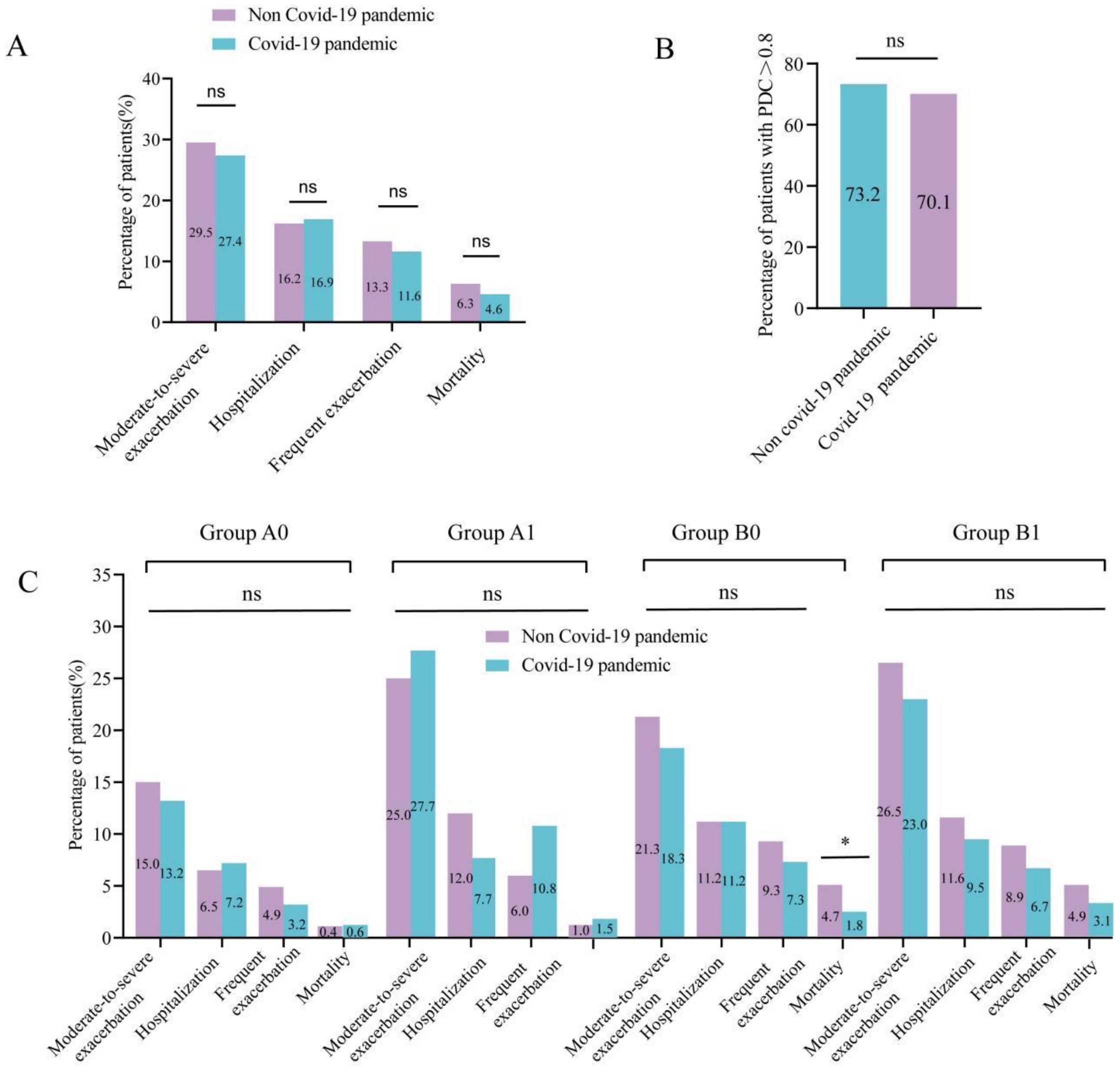

Supplemental Table 2. Hazard ratios for exacerbation and mortality for group A1, B0, B1, E, versus A0.

	Moderate-to-severe exacerbation		Hospitalization		Frequent exacerbation		Mortality	
	HR (95CI%)	*P*-value	HR (95CI%)	*P*-value	HR (95CI%)	*P*-value	HR (95CI%)	*P*-value
A0	Reference		Reference		Reference		Reference	
A1	1.897 (1.407-2.891)	**0.001**	1.512 (1.002-2.117)	**0.048**	1.319 (1.001-1.823)	**0.031**	1.967 (0.416-9.649)	0.386
B0	1.376 (1.098-1.723)	**0.006**	1.647 (1.004-2.323)	**0.042**	1.515 (1.013-2.079)	**0.021**	2.746 (1.207-6.247)	**0.016**
B1	2.013 (1.331-2.983)	< **0.001**	1.679 (1.005-2.411)	**0.040**	1.589 (1.008-2.112)	**0.028**	3.168 (1.330-6.543)	**0.006**
E	3.897 (3.196-4.752)	< **0.001**	3.817 (2.940-4.954)	< **0.001**	5.129 (3.716-7.078)	**0.005**	3.361 (1.499-6.535)	**0.003**

Notes: Age, gender, smoking status, FEV1%, CAT, PDC were included as the variables in the multivariate Cox analysis. **Abbreviations:** GOLD, Global Initiative for Chronic Obstructive Lung Disease; HR, hazard risk, COVID-19,coronavirus disease 2019; PDC, proportion of days covered.

.

Supplemental Table 3. Hazard ratios of different inhalation drug for future exacerbation and mortality in GOLD B0,B1.

		Moderate-to-severe exacerbation		Hospitalization		Frequent exacerbation		Mortality	
		HR (95CI%)	*P*-value	HR (95CI%)	*P*-value	HR (95CI%)	*P*-value	HR (95CI%)	*P*-value
B0	LABA	Reference		Reference		Reference		Reference	
	ICS+LABA	1.010 (0.764-1.3334)	0.947	1.065 (0.745-1.524)	0.729	1.318 (0.992-1.637)	0.052	1.631 (0.916-2.301)	0.063
	LABA+LAMA	0.647 (0.439-0.954)	0.028	0.644 (0.385-1.077)	0.096	0.743 (0.420-1.314)	0.307	0.436 (0.118-1.618)	0.215
	ICS+LABA+LAMA	0.939 (0.724-1.218)	0.636	1.011 (0.723-1.413)	0.950	0.840 (0.568-1.241)	0.381	1.898 (0.946-2.805)	0.071
B1	LABA	Reference		Reference		Reference		Reference	
	ICS+LABA	1.485 (0.978-2.256)	0.063	1.618 (0.906-2.889)	0.104	1.263 (0.668-1.386)	0.473	1.609 (0.704-2.542)	0.239
	LABA+LAMA	0.584 (0.342-0.999)	0.050	0.749 (0.348-1.631)	0.460	0.577 (0.242-1.376)	0.215	0.708 (0.203-2.382)	0.572
	ICS+LABA+LAMA	0.669 (0.461-1.062)	0.093	0.871 (0.479-1.584)	0.651	0.643 (0.329-1.260)	0.198	0.867 (0.345-2.179)	0.762

Notes: Age, gender, smoking status, FEV1%, CAT, PDC were included as the variables in the multivariate Cox analysis. **Abbreviations:** GOLD, Global Initiative for Chronic Obstructive Lung Disease; HR, hazard risk; ICS, inhaled corticosteroids; LABA, long-acting β-2-agonist; LAMA, long-acting muscarinic antagonist,COVID-19,coronavirus disease 2019; PDC, proportion of days covered.

Supplemental Table 4. Hazard ratios for future exacerbation and mortality for GOLD A1 versus A0 during COVID-19 pandemic.

	**Moderate-to-severe exacerbation**		**Hospitalization**		**Frequent exacerbation**		**Mortality**	
	HR (95CI%)	*P*-value	HR (95CI%)	*P*-value	HR (95CI%)	*P*-value	HR (95CI%)	*P*-value
A0	Reference		Reference		Reference		Reference	
A1	1.607 (1.366-2.785)	**0.004**	0.656 (0.193-2.233)	0.500	2.027 (1.037-3.841)	**0.043**	1.332 (0.889-2.352)	0.285

Notes: Age, gender, education, BMI, smoking status, comorbidities, FEV1%, CAT, PDC were included as the variables in the multivariate Cox analysis.
Abbreviations: GOLD, Global Initiative for Chronic Obstructive Lung Disease; HR, hazard risk,COVID-19,coronavirus disease 2019; PDC, proportion of days covered.

Supplemental Table 5. Hazard ratios for future exacerbation and mortality for GOLD B1 versus B0 during COVID-19 pandemic.

	**Moderate-to-severe exacerbation**		**Hospitalization**		**Frequent exacerbation**		**Mortality**	
	HR (95CI%)	*P*-value	HR (95CI%)	*P*-value	HR (95CI%)	*P*-value	HR (95CI%)	*P*-value
B0	Reference		Reference		Reference		Reference	
B1	1.212 (1.008-1.904)	**0.012**	0.795 (0.475-1.333)	0.385	1.045 (0.585-1.865)	0.884	1.593 (1.059-2.925)	**0.030**

Notes: Age, gender, education, BMI, smoking status, comorbidities, FEV1%, CAT, PDC were included as the variables in the multivariate Cox analysis.
Abbreviations: GOLD, Global Initiative for Chronic Obstructive Lung Disease; HR, hazard risk,COVID-19,coronavirus disease 2019; PDC, proportion of days covered.

Supplemental Table 6. Hazard ratios for future exacerbation and mortality for GOLD A1 versus A0 during non-COVID-19 pandemic.

	Moderate-to-severe exacerbation		Hospitalization		Frequent exacerbation		Mortality	
	HR (95CI%)	*P*-value	HR (95CI%)	*P*-value	HR (95CI%)	*P*-value	HR (95CI%)	*P*-value
A0	Reference		Reference		Reference		Reference	
A1	1.441 (1.066-2.776)	**0.015**	1.458 (1.024-2.903)	**0.044**	0.758 (0.167-2.432)	0.719	1.015 (0.814-1.738)	0.871

Notes: Age, gender, education, BMI, smoking status, comorbidities, FEV1%, CAT, PDC were included as the variables in the multivariate Cox analysis.

Abbreviations: GOLD, Global Initiative for Chronic Obstructive Lung Disease; HR, hazard risk,COVID-19,coronavirus disease 2019; PDC, proportion of days covered.

Supplemental Table 7. Hazard ratios for future exacerbation and mortality for GOLD B1 versusB0 during non-COVID-19 pandemic.

	**Moderate-to-severe exacerbation**		**Hospitalization**		**Frequent exacerbation**		**Mortality**	
	HR (95CI%)	*P*-value	HR (95CI%)	*P*-value	HR (95CI%)	*P*-value	HR (95CI%)	*P*-value
B0	Reference		Reference		Reference		Reference	
B1	1.323 (1.073-1.632)	**0.009**	1.017 (0.765-1.353)	0.906	0.944 (0.687-1.296)	0.719	0.938 (0.611-1.440)	0.771

Notes: Age, gender, education, BMI, smoking status, comorbidities, FEV1%, CAT, PDC were included as the variables in the multivariate Cox analysis.

Abbreviations: GOLD, Global Initiative for Chronic Obstructive Lung Disease; HR, hazard risk,COVID-19,coronavirus disease 2019; PDC, proportion of days covered.

Supplemental Table 8. Hazard ratios of different inhalation drug for future exacerbation and mortality in group A0,A1,B0,B1 during COVID-19 pandemic

		Moderate-to-severe exacerbation		Hospitalization		Frequent exacerbation		Mortality	
		HR (95CI%)	*P*-value	HR (95CI%)	*P*-value	HR (95CI%)	*P*-value	HR (95CI%)	*P*-value
A0	LABA	Reference		Reference		Reference		Reference	
	ICS+LABA	0.998 (0.530-1.888)	0.991	0.410 (0.134-1.254)	0.118	1.520 (0.422-2.475)	0.522	1.220 (0.645-2.855)	0.996
	LABA+LAMA	1.560 (0.813-1.994)	0.181	1.522 (0.667-2.471)	0.318	1.668 (0.591-2.705)	0.233	1.593 (0.625-2.341)	0.643
	ICS+LABA +LAMA	1.574 (0.784-2.197)	0.202	1.496 (0.824-2.453)	0.225	1.953 (0.747-3.089)	0.170	0.986 (0.410-1.367)	0.415
A1	LABA	Reference		Reference		Reference		Reference	
	ICS+LABA	0.922 (0.615-1.954)	0.913	0.989 (0.123-3.546)	0.998	0.595 (0.048-3.443)	0.687	0.911 (0.102-3.498)	0.951
	LABA+LAMA	1.433 (0.292-2.035)	0.658	0.962 (0.201-3.115)	0.986	2.024 (0.217-3.848)	0.536	0.678 (0.098-3.123)	0.999
	ICS+LABA +LAMA	0.363 (0.059-2.218)	0.272	0.924 (0.289-3.361)	0.954	1.369 (0.164-2.447)	0.772	0.824 (0.411-3.367)	0.979
B0	LABA	Reference		Reference		Reference		Reference	
	ICS+LABA	1.091 (0.655-1.818)	0.738	1.260 (0.679-2.337)	0.464	0.767 (0.383-1.534)	0.453	1.042 (0.284-3.830)	0.950
	LABA+LAMA	0.652 (0.392-0.982)	**0.038**	0.679 (0.358-1.287)	0.236	0.442 (0.216-0.905)	**0.025**	0.274 (0.051-1.475)	0.132
	ICS+LABA +LAMA	0.978 (0.634-1.105)	0.522	1.090 (0.638-1.859)	0.753	0.457 (0.244-0.855)	**0.014**	0.486 (0.139-1.697)	0.258
B1	LABA	Reference		Reference		Reference		Reference	
	ICS+LABA	0.264 (0.072-0.970)	**0.033**	0.632 (0.352-1.133)	0.123	0.767 (0.383-1.534)	0.453	1.042 (0.284-3.830)	0.950
	LABA+LAMA	0.214 (0.091-0.504)	**＜0.001**	0.237 (0.082-0.682)	**0.008**	0.442 (0.216-0.905)	**0.025**	0.274 (0.051-1.475)	0.132
	ICS+LABA	0.190 (0.080-0.452)	**＜0.001**	0.058 (0.012-0.285)	**＜0.001**	0.457 (0.244-0.855)	**0.014**	0.486 (0.139-1.697)	0.258

+LAMA

Notes: Age, gender, education, BMI, smoking status, comorbidities, FEV1%, CAT, PDC were included as the variables in the multivariate Cox analysis.

Abbreviations: GOLD, Global Initiative for Chronic Obstructive Lung Disease; HR, hazard risk,COVID-19,coronavirus disease 2019; PDC, proportion of days covered.

Supplemental Table 9. Hazard ratios of different inhalation drug for future exacerbation and mortality in group A0,A1,B0,B1 during non-COVID-19 pandemic

		Moderate-to-severe exacerbation		Hospitalization		Frequent exacerbation		Mortality	
		HR (95CI%)	*P*-value	HR (95CI%)	*P*-value	HR (95CI%)	*P*-value	HR (95CI%)	*P*-value
A0	LABA	Reference		Reference		Reference		Reference	
	ICS +LABA	0.927 (0.540-1.589)	0.782	0.941 (0.392-2.256)	0.891	1.161 (0.473-2.849)	0.745	1.112 (0.923-1.355)	0.994
	LABA +LAMA	0.365 (0.125-1.064)	0.065	0.802 (0.223-2.882)	0.735	1.013 (0.901-1.203)	0.997	0.995 (0.923-1.101)	0.999
	ICS +LABA +LAMA	0.986 (0.552-1.763)	0.962	1.667 (0.765-3.678)	0.197	1.636 (0.655-4.084)	0.292	0.986 (0.933-1.198)	0.994
A1	LABA	Reference		Reference		Reference		Reference	
	ICS +LABA	0.719 (0.237-2.180)	0.560	1.114 (0.275-3.250)	0.880	1.889 (0.213-4.102)	0.425	1.230 (0.230-4.056)	0.998
	LABA +LAMA	0.488 (0.123-1.939)	0.308	0.873 (0.173-3.402)	0.870	1.430 (0.536-2.440)	0.142	0.875 (0.121-4.214)	0.999
	ICS +LABA +LAMA	0.209 (0.040-0.988)	**0.043**	0.310 (0.033-2.964)	0.310	0.997 (0.364-3.489)	0.999	0.824 (0.345-4.539)	0.998
B0	LABA	Reference		Reference		Reference		Reference	
	ICS +LABA	0.957 (0.685-1.339)	0.800	0.798 (0.509-1.251)	0.325	0.623 (0.385-1.007)	0.054	0.271 (0.107-1.804)	**0.008**
	LABA +LAMA	0.793 (0.410-1.553)	0.490	0.646 (0.163-1.320)	0.150	0.719 (0.299-1.731)	0.462	0.222 (0.029-1.694)	0.147
	ICS +LABA +LAMA	0.909 (0.704-1.174)	0.466	0.905 (0.653-1.255)	0.551	0.597 (0.421-0.848)	**0.004**	0.902 (0.565-1.440)	0.666
**B1**	LABA	Reference		Reference		Reference		Reference	
	ICS +LABA	0.765 (0.478-1.224)	0.264	0.767 (0.396-1.488)	0.433	0.907 (0.443-1.855)	0.789	0.910 (0.353-2.351)	0.846
	LABA +LAMA	0.507 (0.214-0.985)	**0.039**	0.527 (0.143-0.99	**0.045**	0.504 (0.143-1.783)	0.288	0.302 (0.037-2.463	0.264

			6)				)	
ICS +LABA +LAMA	0.600 (0.386-0.934)	**0.024**	0.492 (0.201-0.991)	**0.023**	0.746 (0.378-0.997)	**0.043**	0.901 (0.364-2.235)	0.823

Notes: Age, gender, education, BMI, smoking status, comorbidities, FEV1%, CAT, PDC were included as the variables in the multivariate Cox analysis.

Abbreviations: GOLD, Global Initiative for Chronic Obstructive Lung Disease; HR, hazard risk,COVID-19,coronavirus disease 2019; PDC, proportion of days covered.
